# RETRACTION: C/EBP*α*‐Mediated Transcriptional Activation of PIK3C2A Regulates Autophagy, Matrix Metalloproteinase Expression, and Phenotypic of Vascular Smooth Muscle Cells in Aortic Dissection

**DOI:** 10.1155/jimr/9860463

**Published:** 2026-10-13

**Authors:** Journal of Immunology Research

RETRACTION: H. Lu, Y. Chen, Y. Chen, L. Huang, and L. Chen, “C/EBP*α*‐Mediated Transcriptional Activation of PIK3C2A Regulates Autophagy, Matrix Metalloproteinase Expression, and Phenotypic of Vascular Smooth Muscle Cells in Aortic Dissection,” *Journal of Immunology Research* 2022, no. 1 (2022): 7465353, https://doi.org/10.1155/2022/7465353.

The above article, published online on 12 September 2022 in Wiley Online Library (wileyonlinelibrary.com), has been retracted by agreement between the Journal Editor‐in‐Chief, Nadja Bakocevic and John Wiley & Sons Ltd. The retraction has been agreed after the authors initially contacted the journal requesting to replace the Western Blot images due to using the incorrect antibody.

Further investigation noted unexpected similarities between the GADPH bands in Figure 7a,b. The bands depicting ‘a‐SMA’, in Figure 7a, and ‘SM‐MHC’, in Figure 11, also show hallmarks suggesting image manipulation.

The authors were unresponsive to the journal’s request for their raw data. As such, the Chief Editor no longer considers the results to be reliable, and this article is therefore retracted.

